# *Gata6*-Dependent GLI3 Repressor Function is Essential in Anterior Limb Progenitor Cells for Proper Limb Development

**DOI:** 10.1371/journal.pgen.1006138

**Published:** 2016-06-28

**Authors:** Shinichi Hayashi, Ryutaro Akiyama, Julia Wong, Naoyuki Tahara, Hiroko Kawakami, Yasuhiko Kawakami

**Affiliations:** 1 Department of Genetics, Cell Biology and Development, University of Minnesota, Minneapolis, Minnesota, United States of America; 2 Stem Cell Institute, University of Minnesota, Minneapolis, Minnesota, United States of America; University of Florida, UNITED STATES

## Abstract

*Gli3* is a major regulator of Hedgehog signaling during limb development. In the anterior mesenchyme, GLI3 is proteolytically processed into GLI3R, a truncated repressor form that inhibits Hedgehog signaling. Although numerous studies have identified mechanisms that regulate *Gli3* function in vitro, it is not completely understood how *Gli3* function is regulated in vivo. In this study, we show a novel mechanism of regulation of GLI3R activities in limb buds by *Gata6*, a member of the GATA transcription factor family. We show that conditional inactivation of *Gata6* prior to limb outgrowth by the *Tcre* deleter causes preaxial polydactyly, the formation of an anterior extra digit, in hindlimbs. A recent study suggested that *Gata6* represses *Shh* transcription in hindlimb buds. However, we found that ectopic Hedgehog signaling precedes ectopic *Shh* expression. In conjunction, we observed *Gata6* and *Gli3* genetically interact, and compound heterozygous mutants develop preaxial polydactyly without ectopic *Shh* expression, indicating an additional prior mechanism to prevent polydactyly. These results support the idea that *Gata6* possesses dual roles during limb development: enhancement of *Gli3* repressor function to repress Hedgehog signaling in the anterior limb bud, and negative regulation of *Shh* expression. Our in vitro and in vivo studies identified that GATA6 physically interacts with GLI3R to facilitate nuclear localization of GLI3R and repressor activities of GLI3R. Both the genetic and biochemical data elucidates a novel mechanism by *Gata6* to regulate GLI3R activities in the anterior limb progenitor cells to prevent polydactyly and attain proper development of the mammalian autopod.

## Author Summary

*Gli3* is a major regulator of Hedgehog signaling in the limb, where *Gli3* counteracts Sonic hedgehog (*Shh*) for patterning and proliferative expansion of limb progenitor cells. In the anterior limb mesenchyme, GLI3 is proteolytically processed into GLI3R, a truncated repressor form that inhibits Hedgehog signaling. In this study, we show a novel mechanism of regulation of GLI3R activities in limb buds by *Gata6*, a member of GATA transcription factor family. Conditional inactivation of *Gata6* in mice caused formation of an extra digit in the anterior hindlimbs, a common congenital limb malformation. This phenotype was associated with ectopic Hedgehog signaling activation, and later ectopic *Shh* expression, in the anterior of hindlimb buds. We show that *Gata6; Gli3* compound heterozygous mutants developed anterior extradigit without ectopic *Shh* expression, indicating there to be an additional and prior mechanism before ectopic *Shh* activation that induces extradigit formation. We identified that GATA6 physically interacts with GLI3R and that the interaction facilitates nuclear localization of GLI3R and repressor activities of GLI3R. Therefore, our study identified a novel mechanism by *Gata6* to regulate GLI3R activities in the anterior limb mesenchyme to prevent extra digit formation and proper development of the mammalian autopod.

## Introduction

Understanding the developmental mechanisms that regulate progenitor cells to generate organs with specific morphology and function is a central topic in developmental biology. The vertebrate limb has been serving as an excellent system for such studies. In particular, mesenchymal progenitor cells in limb buds are specified, patterned and expanded to generate each skeletal element with a distinct morphology at each defined position to create the stereotypical limb skeletal system. The mammalian autopod possesses five digits, termed as d1-d5, in an anterior to posterior order. The number and identity of digits have been used as a readout of specification, patterning, and proliferative expansion of progenitor cells [1].

*Sonic Hedgehog* (*Shh*) is expressed in the zone of polarizing activity (ZPA), located at the posterior mesenchyme of the limb bud, and acts as a major regulatory molecule for limb development [1, 2]. Anterior-posterior specification of digit progenitors is regulated by the concentration and duration of progenitor exposure to SHH [3–6]. SHH also regulates the proliferative expansion of mesenchymal progenitor cells to generate a sufficient number of cells to develop into cartilage condensations [7, 8]. Accordingly, ectopic expression of *Shh* in the anterior portion is associated with preaxial polydactyly, which is characterized by the formation of ectopic digits in the anterior of the limb [9]. By contrast, the most anterior digit (d1) develops in a SHH-independent manner [10, 11]. Recent studies have shown that anterior genetic programs, such as *Irx3*-*Irx5* and *Sall4*, are required for development of d1, at least in part, by excluding SHH signaling from the anterior mesenchyme [12, 13].

The glioma-associated oncogene family (GLI) proteins are zinc finger DNA binding proteins, which play diverse roles in animal development and diseases [14]. Among the three *Gli* genes, *Gli3* encodes a bi-functional molecule, acting as both an activator (GLI3A) and a repressor (GLI3R), whose balance depends on Hedgehog signaling [14]. In the presence of Hedgehog ligands, its signal transduction at primary cilia causes inhibition of proteolytic processing of GLI3 [15]. This results in the accumulation of a full-length activator form of GLI3 (GLI3A) in the posterior mesenchyme. In contrast, in the absence of Hedgehog signaling, GLI3 is subjected to proteolysis, generating a truncated repressor form (GLI3R), which accumulates in the anterior mesenchyme. Because GLI1 lacks a repressor domain and GLI2 predominantly functions as an activator [16, 17], GLI3R is the major GLI repressor in the limb [18].

Consistent with the important function of *Gli3* in limb development, its mutations cause developmental defects in mice and humans [19–21]. In particular, *Gli3*^*-/-*^ mice develop polydactyly [21]. Genetic studies in mice demonstrated that a predominant function of *Gli3* is to repress Hedgehog signaling target genes [22, 23]. Furthermore, it has been shown that the balance of GLI3A and GLI3R regulates digit number and identity [24–26]. Numerous studies have shown that multiple mechanisms regulate GLI3 functions in vitro, such as posttranslational modifications, degradation, cytoplasmic retention, and primary cilium-mediated processing (reviewed in [14, 27, 28]). In vivo studies in mice demonstrated that *Gli3* genetically interacts with *Hox* genes, *Zic3* and *Alx4* during limb development [29–31]. Despite these studies, the in vivo control of *Gli3* function during proper limb development is still to be elucidated.

The *Gata* family of zinc finger transcription factors is an important regulator of tissue and organ development. The *Gata* family is subdivided into the *Gata1/2/3* subfamily and the *Gata4/5/6* subfamily, which show expression in hematopoietic cell lineages and meso-endoderm lineages, respectively [32, 33]. In particular, *Gata6* is essential for endoderm formation and is also involved in the development of various mesoderm- and endoderm-derived organs, such as the cardiovascular system and pancreas [34–37]. Moreover, a recent study suggested that *Gata6* functions as a negative regulator of *Shh* expression in limb buds by binding to its limb bud-specific cis-regulatory element, ZRS [38].

In this study, we found that broad deletion of *Gata6* in the limb mesenchymal progenitors caused hindlimb-specific preaxial polydactyly, which is associated with ectopic SHH signaling in the anterior hindlimb bud. We discovered that *Gata6* and *Gli3* genetically interact to regulate normal patterning of the hindlimb. Furthermore, we show that direct association of GATA6 with GLI3R promoted nuclear localization and transcriptional repressor activity of GLI3R. Our work identified that genetic and biochemical interactions between *Gata6* and *Gli3* act as essential mechanisms to regulate GLI3R activity for proper autopod patterning.

## Results

### Inactivation of *Gata6* in early mesoderm caused hindlimb specific preaxial polydactyly

**Fig 1:**
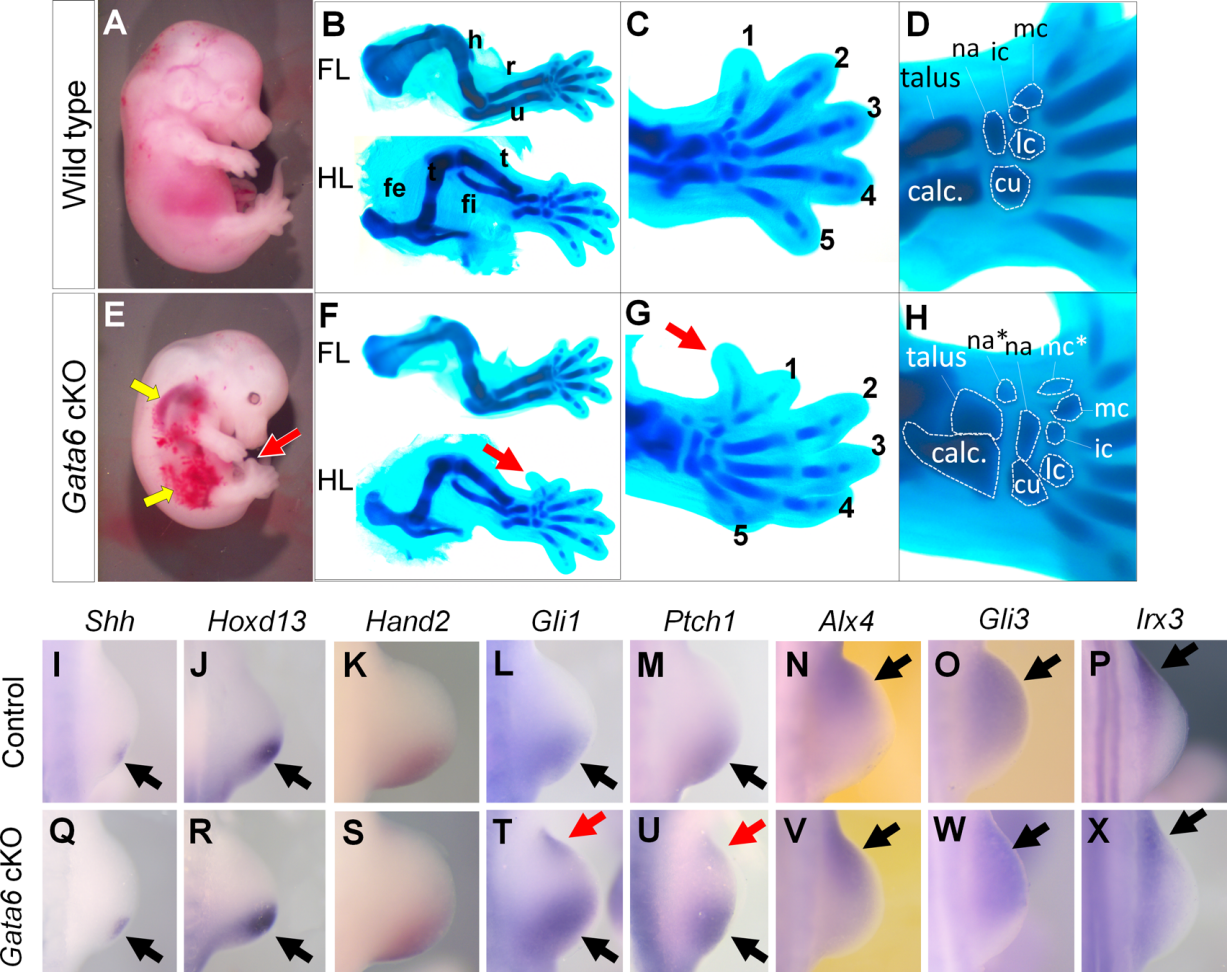
Loss of Gata6 causes preaxial polydactyly in hindlimbs. **A-H**: Lateral views (**A, E**) of whole E14.5 embryos, and Alcian blue-stained cartilage (**B-D, F-H**) of wild type (**A-D**) and *Gata6* cKO (**F-H**) embryos at E14.5. **C** and **G** show hindlimb autopod, and **D** and **H** show tarsal and metatarsal elements. Red arrows in **E-G** point to the anterior ectopic digit. Yellow arrows point to hemorrhage in *Gata6* cKO embryos. Digits are numbered with 1–5 in **C** and **G**. Asterisks in **H** indicates ectopic elements. calc: calcaneus, cu: cuboid, fe: femur, fi: fibula, ic: intermediate cuneiform, lc: lateral cuneiform, mc: medial cuneiform, na: navicular ti: tibia. **I-X**: in situ hybridization of wild type (**I-P**) and *Gata6* cKO (**Q-X**) hindlimb buds at E10.5 with indicated probes. Black and red arrows point to normal and ectopic signals, respectively. See also S1 Table.

Prior studies have identified expression of *Gata6* in developing limb buds [38–40]. *Gata6* null embryos die during gastrulation [34, 35]; therefore, we inactivated *Gata6* in the meso-endoderm by using the conditional allele of *Gata6* (*Gata6*^*fl*^) [41] and the *Tcre* line, which recombines in the early meso-endoderm [42]. We found that *Tcre; Gata6*^*fl/fl*^ mutants (hereafter referred to as *Gata6* cKO) die around E12.5–14.5 with broad hemorrhage (Fig 1A and 1E). This result is consistent with a former study, demonstrating a role of proper dosage of *Gata4* and *Gata6* for vessel integrity [43]. We found that *Gata6* cKO embryos exhibited polydactyly in the hindlimb, while forelimbs seem to be unaffected (Fig 1A–1C, 1E and 1G, S1 Table). Alcian blue staining demonstrated that the mutant hindlimbs possess patterned digits, d1-d5, and an extra digit on the anterior edge, which morphologically resembles d1. Based on the position and morphology, tarsal and metatarsal elements were also patterned. Two ectopic tarsal elements, likely the navicular and medial cuneiform, were present proximally to the ectopic 1^st^ metatarsal (Fig 1D and 1H). These observations indicate that the autopod is patterned along the anterior-posterior axis, and the absence of *Gata6* induces the formation of an extra anterior digit with the associated tarsal and metatarsal elements.

### Ectopic Hedgehog signaling activation in *Gata6* cKO hindlimbs

Preaxial polydactyly is known to be associated with ectopic *Sonic Hedgehog* (*Shh*) expression in the anterior margin. At E10.5, we detected posteriorly-localized *Shh* expression without ectopic anterior expression (n = 4, 39–40 somite stage, Fig 1I and 1Q). Consistent with this normal expression, *Hoxd13* (n = 3) and *Hand2* (n = 6), upstream regulators of limb bud *Shh* expression [44], were normally expressed in the posterior mesenchyme (Fig 1J, 1K, 1R and 1S). However, *Gli1* (n = 3) and *Patch1* (n = 3), targets of Hedgehog signaling, were detected in the anterior margin of *Gata6* cKO hindlimb buds (Fig 1L, 1M, 1T and 1U). Expression of anterior marker genes, such as *Alx4* (n = 3), *Gli3* (n = 4) and *Irx3* (n = 3), were not significantly affected in *Gata6* cKO hindlimb buds (Fig 1N–1P and 1V–1X).

We also examined gene expression at a later stage. At E11.5, we detected ectopic *Shh* expression in the anterior border of *Gata6* cKO hindlimbs (n = 4, S1 Fig). Consistent with evident ectopic *Shh* expression, expression of *Hoxd13* (n = 3), *Gli1* (n = 6), *Ptch1* (n = 6) and *Gremlin1* (n = 3) was also detected in the anterior margin. This data indicates that ectopic Hedgehog signaling became evident at E10.5 in *Gata6* cKO hindlimb buds, although ectopic *Shh* expression was undetectable. At a later stage (E11.5), ectopic *Shh* expression became evident and all SHH targets, examined in this study, were detected in the anterior margin.

*Shh* expression is negatively regulated in the anterior margin by various genes. Thus, we examined expression of negative regulators of *Shh* expression. In addition to *Alx4* and *Gli3* (Fig 1) [23, 45], expression of *Etv4* (n = 3), *Etv5* (n = 5), *Tulp3* (n = 3), *Twist1* (n = 3), whose loss can cause ectopic *Shh* expression in the anterior margin [46–52], did not show evident alteration (S2 Fig). Therefore, it is unlikely that these genes account for the preaxial polydactyly phenotype in *Gata6* cKO hindlimbs.

### Reduction of *Shh* dosage rescued ectopic SHH signaling but not ectopic anterior digit formation

If ectopic *Shh* expression accounts for the preaxial polydactyly in *Gata6* cKO hindlimbs, we would expect that reducing *Shh* dosage might rescue the phenotype. Therefore, we genetically reduced *Shh* dosage from the *Gata6* cKO background using the *Shh* null allele [2]. *Gata6* cKO; *Shh*^+/-^ mutants did not survive beyond E12.5, thus, we examined expression of SHH target genes (*Gli1* and *Ptch1*) and expression of *Sox9*, an early marker of chondrogenic condensation [53].

**Fig 2:**
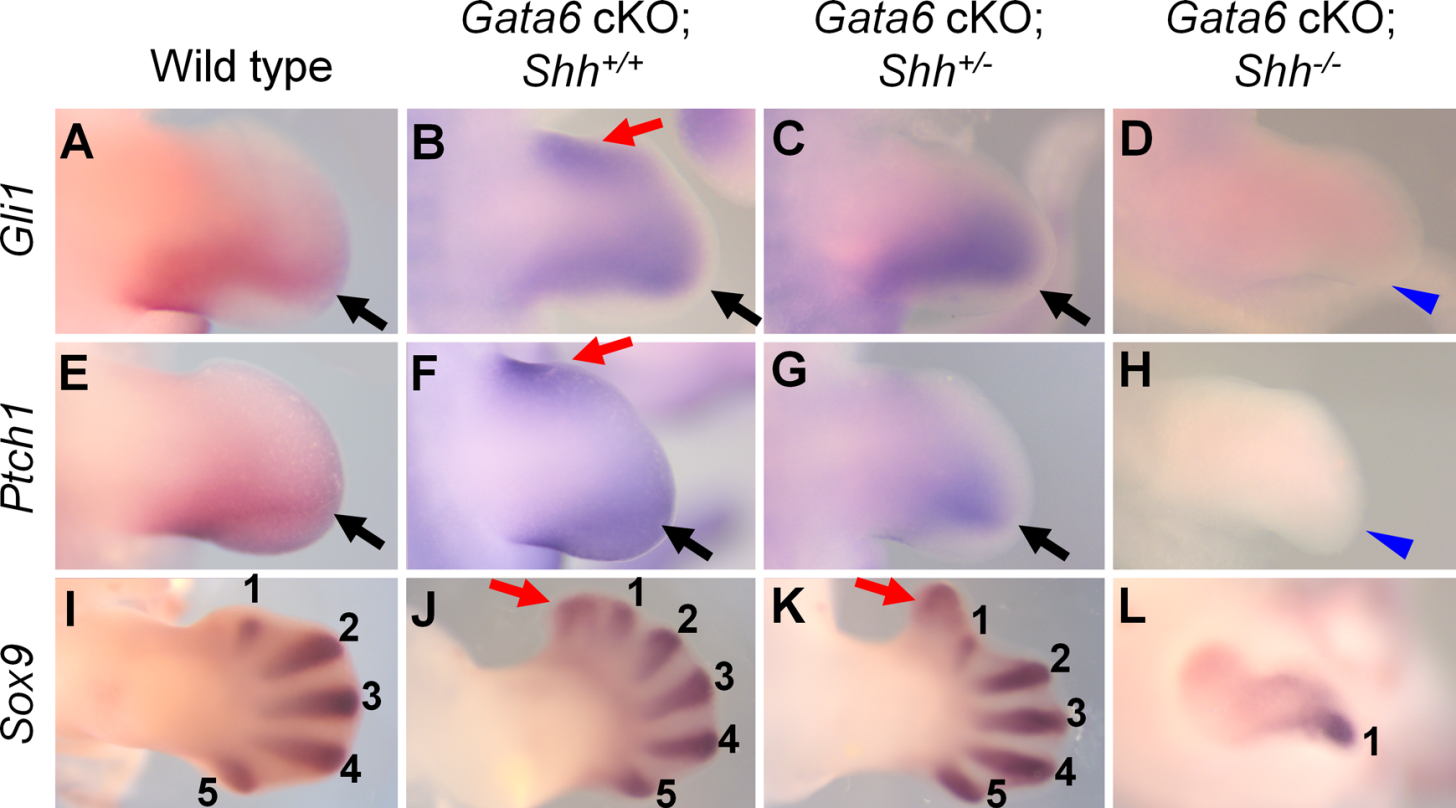
Expression pattern of SHH targets and digit condensation in *Gata6* cKO; *Shh* allelic series. Expression pattern of *Gli1* (**A-D**), *Ptch1* (**E-H**) and *Sox9* (**I-L**) of wild type (**A, E, I**), *Gata6* cKO (**B, F, J**), *Gata6* cKO; *Shh*^*+/-*^ (**C, G, K**) and *Gata6* cKO; *Shh*^*-/-*^ (**D, H, L**) hindlimb buds. **A-H**: E11.5, **I-L**: E12.5. In **A**-**H**, black arrows and red arrows point to normal and ectopic signals, respectively. Blue arrowheads indicate loss of expression in **D** and **H**. In **I-L**, digit condensations are labeled as 1–5, and ectopic condensation is marked with red arrows.

Removing one allele of *Shh* from the *Gata6* cKO background resulted in posteriorly restricted expression of *Gli1* and *Ptch1*, and the ectopic anterior expression became undetectable (n = 4, Fig 2A–2C and 2E–2G). However, ectopic chondrogenic condensation in the anterior portion was still detected by *Sox9* expression at E12.5 (n = 3, Fig 2I–2K). Removing both alleles of *Shh* from the *Gata6* cKO background resulted in the loss of *Gli1* and *Ptch1* expression and single digit condensation, the same phenotype as *Shh*^-/-^ limbs (n = 3, Fig 2D, 2H and 2L) [10, 11]. These results indicate that *Shh* functions downstream of *Gata6* during preaxial polydactyly development. However, ectopic chondrogenic condensation in the anterior portion of *Gata6* cKO; *Shh*^+/-^ hindlimbs suggests that additional mechanisms could be involved in the preaxial polydactyly in *Gata6* cKO hindlimbs.

### *Gli3* genetically interacts with *Gata6* in forelimbs and hindlimbs

**Fig 3:**
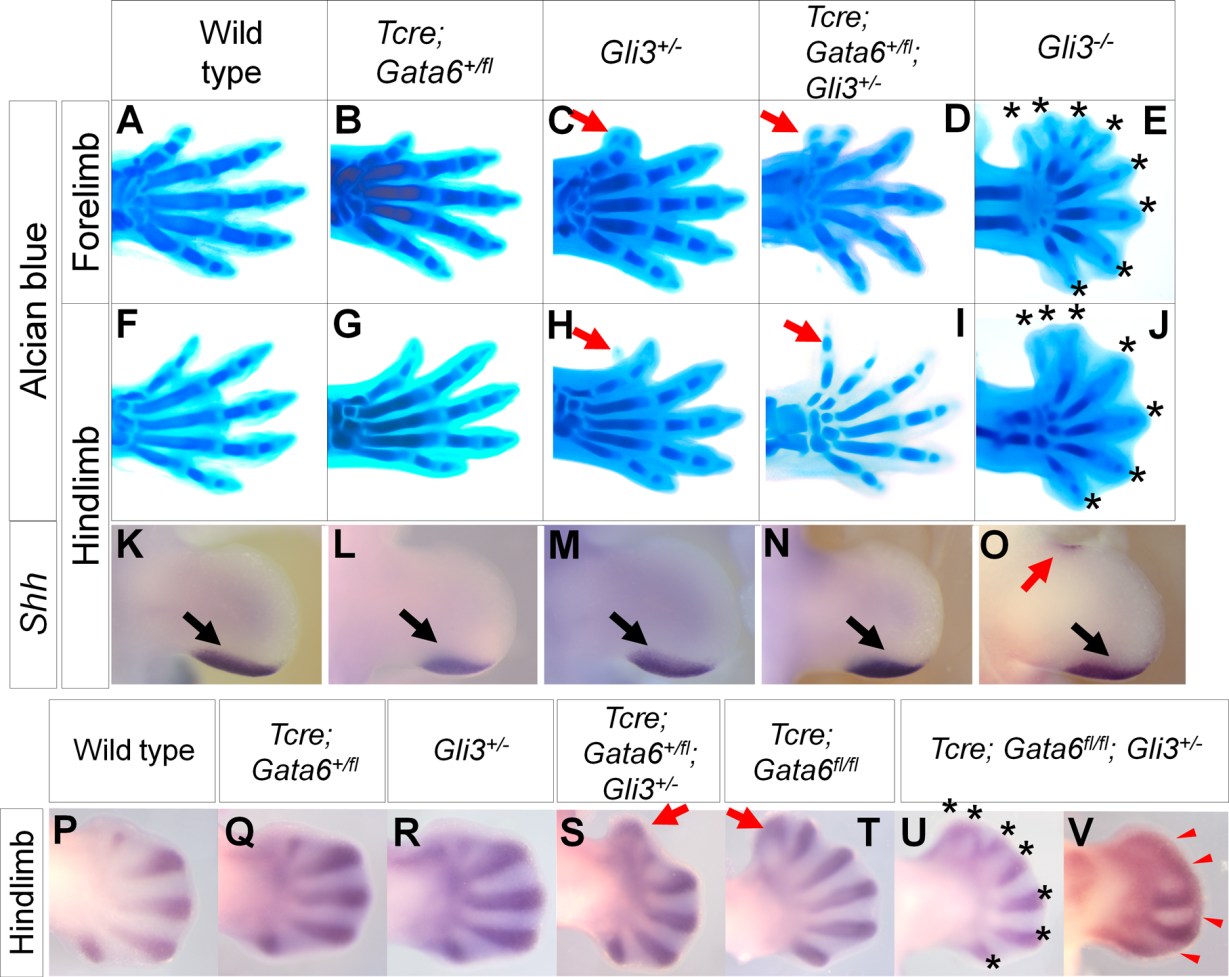
Genetic interaction between *Gata6* and *Gli3* in preaxial polydactyly development. **A-J**: Alcian blue-stained autopod of indicated genotypes at E15.5. A-E: forelimbs, F-J: hindlimbs. Thin red arrows point to bifurcated d1 (**C**) and small projection (**H**) in fore- and hind-limbs, respectively in *Gli3*^*+/-*^ mutants. Thick red arrows in **D** and **I** point to anterior ectopic digits. Asterisks in E and J indicate digit tips of *Gli3*^*-/-*^ autopod. **K-O**: Expression pattern of *Shh* in hindlimb buds of indicated genotypes at E11.5. Black and red arrows point to normal and ectopic signals, respectively. **P-V**: *Sox9* in situ hybridization in hindlimbs of indicated genotypes at E12.5. Red arrows in **S** and **T** point to anterior ectopic digit condensation. Asterisks in **U** indicate distal tips of digit condensation. Red arrowheads in **V** point to distally-fused condensation.

GLI3 is a major regulator of Hedgehog signaling, and thus, *Gli3* might be involved in preaxial polydactyly in *Gata6* cKO hindlimbs. To test this hypothesis, we genetically removed *Gli3* from the *Gata6* cKO background. *Gli3*^*+/-*^ hindlimbs developed a small spike in the anterior region [21, 54], while most of the *Tcre; Gata6*^*+/fl*^ hindlimbs were indistinguishable from the wild-type hindlimbs at E14.5–15.5 (Fig 3F–3H, Table 1). *Tcre; Gata6*^*+/fl*^; *Gli3*^*+/-*^ compound heterozygous hindlimbs developed an extra digit in the anterior region (Fig 3I). Unexpectedly, we also found that this interaction operates in forelimbs. *Gli3*^*+/-*^ forelimbs developed d1, which was associated with small ectopic cartilage condensation at the distal tip. Contrary to this, *Tcre; Gata6*^*+/fl*^*; Gli3*^*+/-*^ compound heterozygous forelimbs developed an evident extra digit with incomplete penetrance (Fig 3A–3D) or an extra digit that partially fused with endogenous d1 with incomplete penetrance (S2 Table). These results demonstrate a genetic interaction between *Gli3* and *Gata6* in fore- and hind-limbs.

**Table 1:** Number of hindlimbs with indicated phenotypes at E14.5–16.5.

Genotype	Number of hindlimbs with normal digits	Number of hindlimbs with small projection	Number of hindlimbs with anterior extra digit
Wild type	140/140 (100%)	0/140	0/140
*Gli3*^*+/-*^	2/18 (11.1%)	16/18 (88.9%)	0/18 (0%)
*Tcre; Gata6*^*+/fl*^	61/66 (92.4%)	0/66 (0%)	5/66 (7.6%)
*Tcre; Gata6*^*+/fl*^*; Gli3*^*+/-*^	3/54 (5.6%)	0/54 (0%)	51/54 (94.4%)

Because the *Gata6* cKO limb phenotype was evident in hindlimbs, we focused the following analysis on hindlimbs. Ectopic *Shh* expression can cause preaxial polydactyly, therefore, we examined *Shh* expression at E11.5. We detected a small domain of anterior ectopic *Shh* expression in *Gli3*^*-/-*^ hindlimbs (n = 3/6, Fig 3O), as previously reported [23]. By contrast, *Tcre; Gata6*^*+/fl*^; *Gli3*^*+/-*^ compound heterozygous hindlimbs did not exhibit anterior ectopic *Shh* expression (n = 6), similar to wild-type, *Tcre; Gata6*^*+/fl*^ (n = 6) and *Gli3*^*+/-*^ (n = 6) hindlimb buds (Fig 3K–3N). Therefore, preaxial polydactyly in *Tcre; Gata6*^*+/fl*^; *Gli3*^*+/-*^ compound heterozygous limbs were unlikely to be caused by ectopic *Shh* expression. Given that GLI3R prevents ectopic digit formation in the anterior portion [55], these results suggest that an interaction between *Gata6* and *Gli3* contributes to GLI3R activities.

*Gata6* cKO; *Gli3*^*+/-*^ embryos do not survive beyond E12.5, therefore, we further examined the interaction between *Gata6* and *Gli3* by visualizing digit condensation by *Sox9* in situ hybridization. Both *Gli3*^*+/-*^ and *Tcre; Gata6*^*+/fl*^ hindlimbs exhibited similar expression patterns to wild-type hindlimbs at E12.5 (Fig 3P–3R). Correlating with preaxial polydactyly at E15.5, *Gata6* cKO and *Tcre; Gata6*^*+/fl*^; *Gli3*^*+/-*^ compound heterozygous hindlimbs exhibited ectopic anterior digit condensation (Fig 3S and 3T). *Gata6* cKO; *Gli3*^*+/-*^ hindlimbs were slightly underdeveloped and exhibited seven digit condensations (n = 2/6, Fig 3U), distally-fused condensation (n = 2/6, Fig 3V) or one extra anterior condensation, similar to *Gata6* cKO hindlimbs (n = 2/6). Formation of multiple extra digits and distal fusion of cartilage condensation are characteristics of *Gli3*^*-/-*^ limbs [21]. Therefore, we speculate that the *Gata6* cKO; *Gli3*^*+/-*^ genotype may be in conditions similar to the *Gli3*^*-/-*^ genotype in hindlimbs. These results further support the idea that loss of *Gata6* leads to reduction of GLI3R activities.

**Fig 4:**
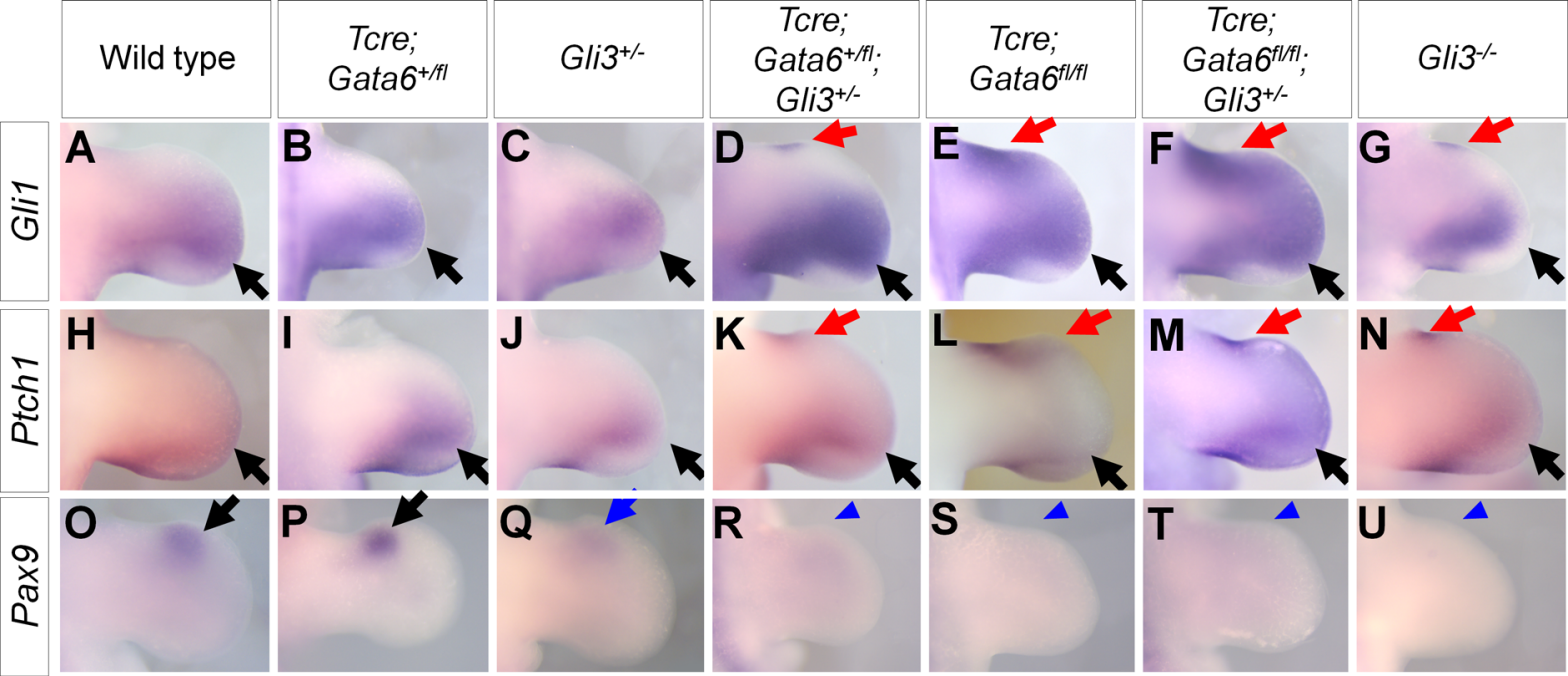
Expression pattern of *Gli1*, *Ptch1* and *Pax9* in *Gata6*; *Gli3* allelic series. In situ hybridization of *Gli1* (**A-G**), *Ptch1* (**H-N**) and *Pax9* (**O-U**) of hindlimb buds of indicated genotypes at E11.5. Black and red arrows point to normal and ectopic signals, respectively. Blue arrows and arrowheads indicate reduced and loss of *Pax9* signals, respectively.

In order to further characterize the *Gata6-Gli3* interaction, we examined gene expression at E11.5. Expression of *Gli1* and *Patch1* was posteriorly restricted in wild-type, *Tcre; Gata6*^*+/fl*^ and *Gli3*^*+/-*^ hindlimbs (Fig 4A–4C and 4H–4J). Hindlimbs with the Tcre; Gata6^+/fl^; Gli3^+/-^, Gata6 cKO, Gata6 cKO; Gli3^+/-^ or Gli3^-/-^ genotypes exhibited anterior ectopic expression of these genes (Fig 4D–4G and 4K–4N). The ectopic expression domain was larger in *Gata6* cKO and *Gata6* cKO; *Gli3*^*+/-*^ hindlimb buds than that in *Tcre; Gata6*^*+/fl*^; *Gli3*^*+/-*^ and *Gli3*^*-/-*^ hindlimbs, likely due to ectopic *Shh* expression in the *Gata6* cKO background.

*Pax9*, whose expression requires high levels of GLI3R activities [56], was detected in the anterior of wild-type and *Tcre; Gata6*^*+/fl*^ hindlimbs, and was reduced in *Gli3*^*+/-*^ hindlimb buds (Fig 4O–4Q). In *Tcre; Gata6*^*+/fl*^; *Gli3*^*+/-*^, *Gata6* cKO, *Gata6* cKO; *Gli3*^*+/-*^ hindlimbs, *Pax9* expression was undetectable, similar to *Gli3*^*-/-*^ hindlimbs (Fig 4R–4U).

These alterations of gene expression at E11.5 are consistent with the idea that GLI3R activities were reduced in hindlimbs with the *Tcre; Gata6*^*+/fl*^; *Gli3*^*+/-*^, *Gata6* cKO and *Gata6* cKO; *Gli3*^*+/-*^ genotypes.

### GATA6 and GLI3 functionally and physically interact in vitro

**Fig 5:**
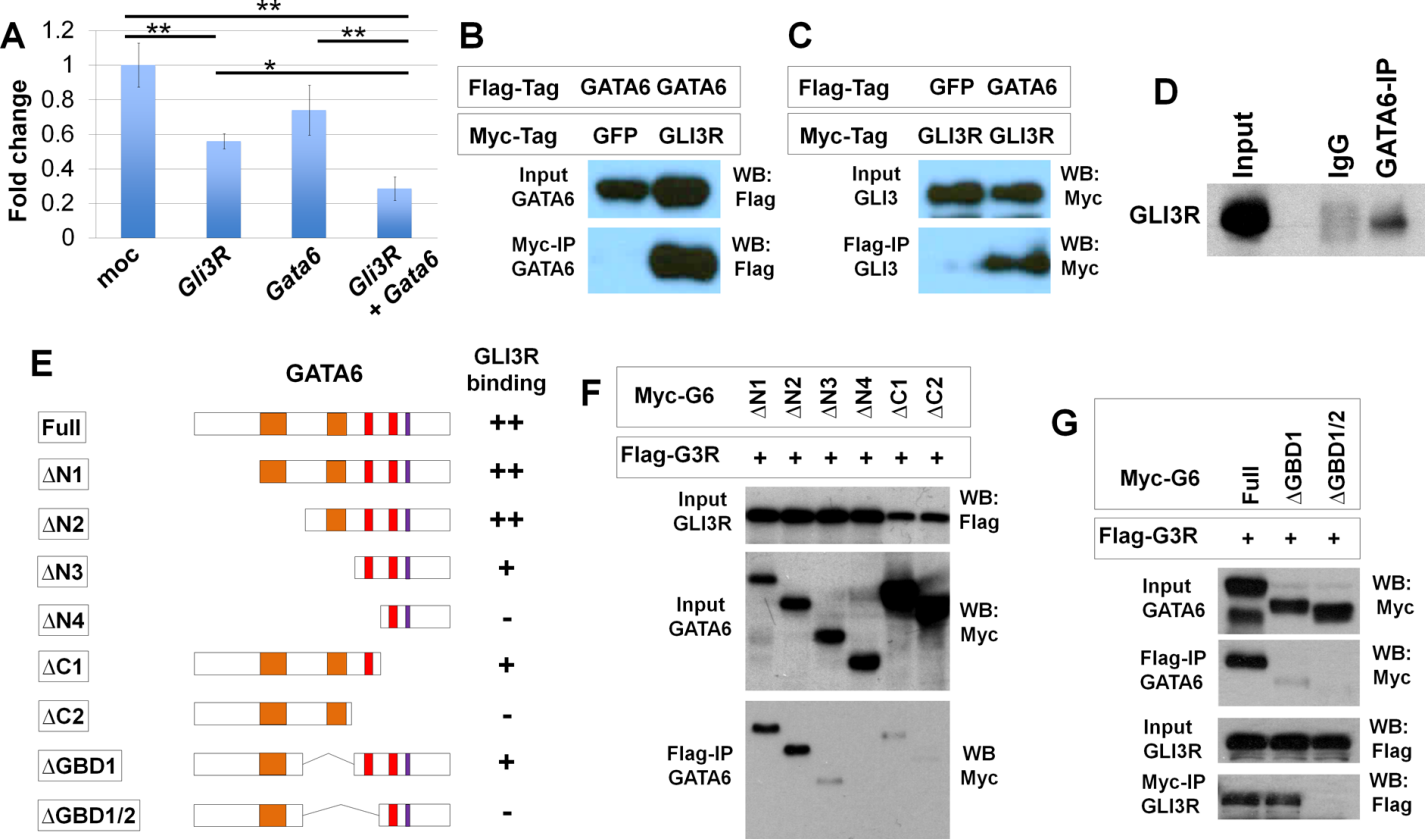
Physical and functional interaction between GATA6 and GLI3R. **A**: GLI-specific luciferase reporter assay with indicated expression constructs. *: p<0.01, **: p<0.001. **B, C**: Co-immunoprecipitation assay of Flag-GATA6 and Myc-GLI3R. (B) Pulldown with anti-Myc, detection by anti-Flag. (C) Pulldown with anti-Flag, detection by anti-Myc. **D**: Co-immunoprecipitation of GATA6 and GLI3R from wild-type hindlimb buds. **E**: Schematic presentation of deletion mutants of GATA6. Binding with GLI3R in **F** and **G** is summarized in the right side of the panel. Orange bars represent transactivation domains. Red and blue bars represent zinc finger DNA binding domains and the nuclear localization signal, respectively. **F, G**: Co-immunoprecipitation assay of Flag-GLI3R and GATA6 mutants.

Ectopic *Shh* expression in the *Gata6* cKO background could affect gene expression patterns in hindlimb buds. Therefore, we set up in vitro experiments to further investigate how *Gata6* regulates *Gli3* function. We first set up luciferase reporter assays using 12xGLI-binding site luciferase [31]. Transfecting a C-terminally truncated form of human *GLI3* that could function as GLI3R caused significant reduction of the reporter activities, while transfecting human *GATA6* did not affect the reporter activities. Co-transfecting *GLI3R* and *GATA6* caused further reduction of the reporter activities (Fig 5A). These results are consistent with the in vivo data and support the idea that *GATA6* functionally interacts with and contributes to GLI3R activities.

Next, we tested whether GATA6 and GLI3R physically interact by co-immunoprecipitation assays. HEK293T cells were transfected with Flag-tagged GATA6, Myc-tagged GLI3R or GFP. Flag-GATA6 and Myc-GLI3R were co-immunoprecipitated, demonstrating that GATA6 and GLI3R can interact (Fig 5B and 5C). We also confirmed that the interaction occurs in vivo. GLI3R was detected in immunoprecipitated complex from E10.25–10.5 wild-type hindlimb buds using ant-GATA6 (Fig 5D). To further characterize their interaction, we mapped the GLI3R interaction domain in GATA6. For this purpose, we generated serial deletion mutants (Fig 5E), and performed co-immunoprecipitation assays with Flag-GLI3R. The ΔN1 and ΔN2 mutants showed a strong interaction with Flag-GLI3R. The ΔN3 and ΔC1 mutants exhibited weak interaction, and we did not detect any interactions of Flag-GLI3R with ΔN4 and ΔC2 (Fig 5F).

We also generated intra-molecular deletion mutants. These mutants lack the GLI3R-binding domain (GBD) 1, which includes the second putative transactivation domain (ΔGBD1), or both GBD1 and GBD2 (ΔGBD1/2). We did not detect any interaction of ΔGBD1/2 with GLI3R, although ΔGBD1 exhibited a weak interaction with GLI3R (Fig 5G). These results suggest that the zinc finger domain 1 (ZFD1) is critical to interact with GLI3R. The weak interaction of ΔN3, ΔC1 and ΔGBD1, which possess the ZFD1, also suggests that both the N- and C-terminal regions around the ZFD1 contribute to the interaction with GLI3R, in collaboration with the ZFD1.

### Interaction between GATA6 and GLI3R regulates subcellular localization of GLI3R

Our analyses indicated the presence of genetic and physical interactions between *Gata6* and *Gli3*. Given that both GATA6 and GLI3R act as transcription factors, we next examined subcellular localization of these proteins after co-transfecting HEK293T cells with Flag-GLI3R and either full length or mutant forms of Myc-GATA6.

**Fig 6:**
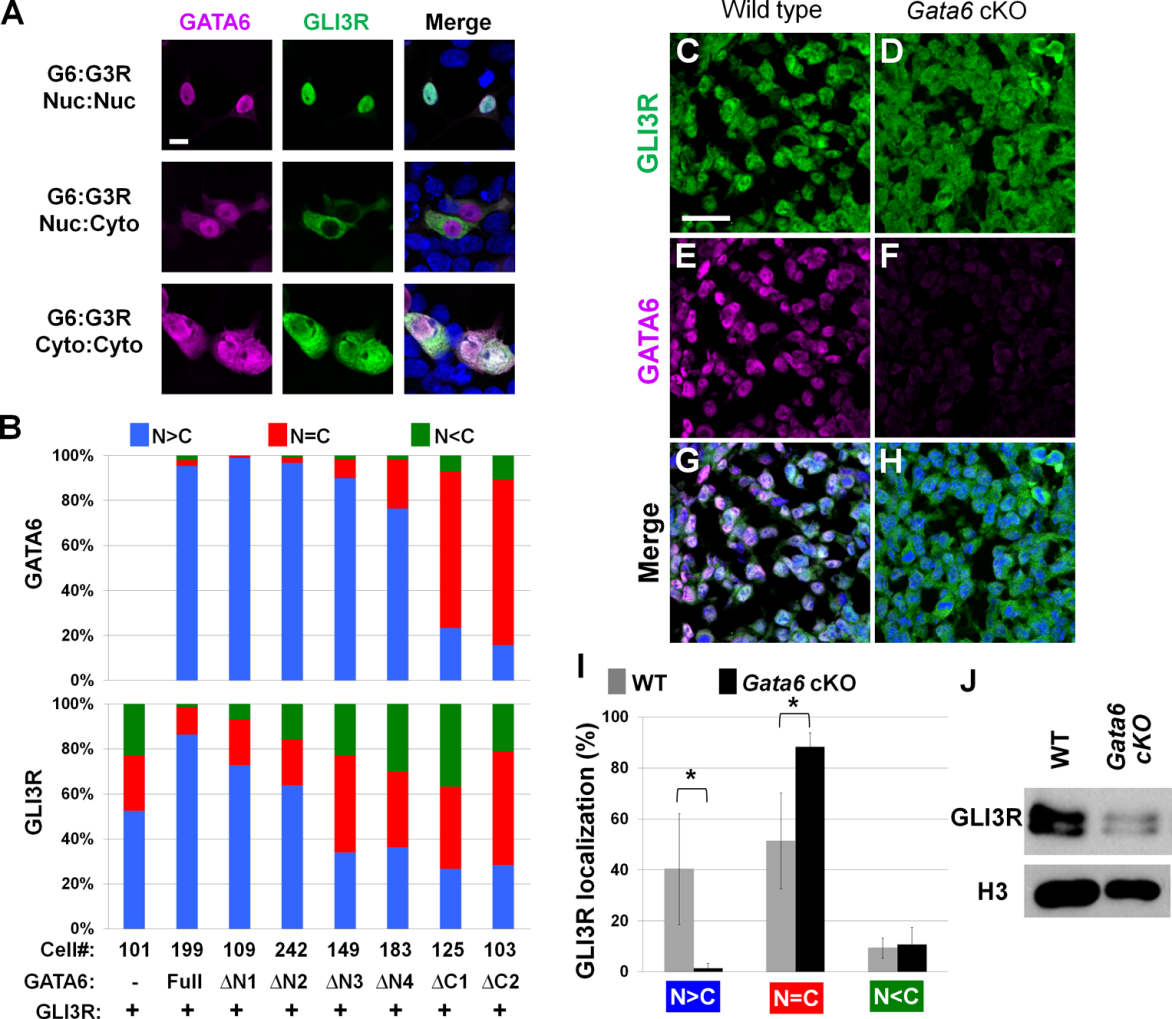
GATA6 regulates subcellular localization of GLI3R. **A**: Representative in vitro images of nuclear GATA6+nuclear GLI3R (upper), nuclear GATA6+cytosolic GLI3R (middle) and cytosolic GATA6+cytosolic GLI3R (bottom). **B**: Quantitation of subcellular localization of GATA6 and GLI3R. N<C: predominantly cytoplasmic, N = C: similarly in cytoplasm and in nucleus, N>C: predominantly nuclear localized. GATA6 mutants, indicated at the bottom, are shown in Fig 5E. The number of cells examined for each set of transfection is indicated in the panel. **C-H**: Representative images of the anterior-proximal mesenchyme of hindlimb buds at E10.25. **C, E, G**: wild type, **D, F, H**: *Gata6* cKO. **I**: Quantitation of subcellular localization of GLI3R in the anterior-proximal mesenchyme of hindlimb buds at E10.25. Gray and black bars represent wild-type and *Gata6* cKO samples, respectively. The graph shows percentage of GLI3R localization patterns, such as predominantly nuclear (N>C), similarly in the nucleus and cytoplasm (N = C), or predominantly cytoplasmic (N<C). A total of 597 cells from three wild-type embryos and a total of 528 cells from three *Gata6* cKO embryos were examined. * indicates P<0.05. **J**: Western blot of nuclear fractions from anterior part of wild-type and *Gata6* cKO hindlimb buds at E10.25–10.5. Histone H3 (H3) is included as a loading control.

We observed three patterns of localization (Fig 6A and 6B, S3 Fig). First, co-transfection of either full length GATA6, ΔN1-GATA6 or ΔN2-GATA6, which can interact with GLI3R and possess the nuclear localization signal (NLS), resulted in predominant nuclear localization of both GLI3R and GATA6. Second, we co-transfected ΔN3-GATA6 or ΔN4-GATA6, which possess the NLS, but have either very weak or undetectable interactions with GLI3R. In these transfection assays, GLI3R localization became either predominantly cytoplasmic or localized similarly in both the cytoplasm and nucleus, although GATA6 was predominantly detected in the nucleus. Third, we co-transfected ΔC1-GATA6 or ΔC2-GATA6, which lack the NLS and have very weak or undetectable interactions with GLI3R. We detected GATA6 predominantly in the cytoplasm, consistent with the lack of NLS. GLI3R was also predominantly located in the cytoplasm or located similarly in the nucleus and cytoplasm.

These results indicate a correlation between GLI3R nuclear localization and nuclear GATA6 that possesses a GLI3R-interaction ability. This correlation suggests that physical association between GATA6 and GLI3R contributes to nuclear localization and the repressor activities of GLI3R. We next tested this idea in vivo by examining GLI3R nuclear localization. The earliest molecular alteration in *Gata6* cKO hindlimb buds in our study is ectopic *Gli1* and *Ptch1* expression at E10.5 (Fig 1). Therefore, we re-examined *Gata6*/GATA6 expression, although their mRNA expression patterns were examined in previous studies [38–40]. *Gata6* mRNA was detected in the anterior-proximal part of hindlimb buds at E10.25 (34 somite stage) (S4A Fig), but the strong signals in endoderm -derived tissues seem to mask the limb bud signals. Therefore, we also performed immunofluorescence of GATA6 in combination with limb bud mesenchyme markers, such as Fibroblast growth factor10 (FGF10) [57] or Dual specificity phosphatase6 (DUSP6) [58–60]. Co-staining with these markers on transverse sections indicates that GATA6 is present in the ventral side of the proximal region in anterior hindlimb buds at E10.25 (S4B and S4C Fig). The GATA6 signal was undetectable in limb buds in the middle-posterior region.

In the anterior proximal region of limb buds at E10.25, we detected GLI3R predominantly in the nucleus or similarly in the nucleus and cytoplasm (Fig 6C, 6E, 6G and 6I). By contrast, *Gata6* cKO hindlimb buds showed a reduced percentage of cells with predominant nuclear GLI3R signals. Accordingly, we detected an increased percentage of cells with nuclear/cytoplasmic GLI3R (Fig 6D, 6H and 6I). Western blot analysis of nuclear extracts from the anterior part of hindlimb buds showed reduced GLI3R levels in *Gata6* cKO, compared to wild-type embryos (Fig 6J). Although the presence of nuclear GLI3R in *Gata6* cKO hindlimb buds indicates *Gata6*-independent GLI3R nuclear localization mechanisms in the anterior mesenchyme, reduced GLI3R levels provide evidence that *Gata6* contributes to GLI3R nuclear localization. These results are consistent with the in vitro data, and further support the idea that *Gata6* regulation of GLI3R nuclear localization contributes to GLI3R activities during normal limb development.

## Discussion

In this study, we found hindlimb-specific preaxial polydactyly in *Gata6* mutants. The skeletal phenotype of *Gata6* mutants was restricted to hindlimbs, and the forelimbs developed normally. Several possibilities would account for such limb type-specific phenotypes. For instance, a recent study showed that *Gata4* is differentially expressed in forelimb buds (high) and hindlimb buds (low) [38]. *Gata4* and *Gata6* are functionally redundant during heart development and for vascular integrity [36, 43]; therefore, *Gata4* might compensate for loss of *Gata6* in forelimb buds [38]. Another possibility is that differences in the sensitivity to Hedgehog signaling contribute to different phenotypes in fore- and hind-limbs. It is suggested that levels of Hedgehog signaling are higher in hindlimb mesenchyme than forelimb mesenchyme [12], and that hindlimbs are more sensitive to changes in the levels of Hedgehog signaling. Higher Hedgehog signaling, in combination with reduced GLI3R, might have contributed to hindlimb-specific polydactyly in *Gata6* cKO. This idea is consistent with ectopic digit formation in *Tcre; Gata6*^*+/fl*^; *Gli3*^*+/-*^ forelimbs, in which GLI3R activities would be lower than and SHH signaling levels would be higher than *Gli3*^*+/-*^ forelimbs. These two scenarios are not mutually exclusive, and they might cooperate together to ensure proper Hedgehog signaling and pentadactyly in mammalian limbs.

Our study proposes two mechanisms by which *Gata6* regulates proper autopod patterning. One mechanism is by enhancing GLI3R activities to repress Hedgehog signaling in the anterior mesenchyme, and the other is by negative regulation of *Shh* expression in the anterior mesenchyme.

Genetic studies have shown that preaxial polydactyly is associated with ectopic expression of *Shh* in the anterior mesenchyme [9]. Expression of *Shh* is positively and negatively regulated in the posterior and anterior mesenchyme, respectively. *Twist1*, *Alx4*, *Gli3*, *Tulp3* and *Etv4*-*Etv5* act as negative regulators, for their loss of function caused ectopic *Shh* expression [23, 47, 49, 51, 61]. Genetic and biochemical studies have shown that *Hand2* and *Hoxd13* positively regulate *Shh* expression through the limb bud-specific cis-regulatory element, ZRS [44, 62]. Anterior *Shh* expression could be induced by loss of negative regulators or ectopic expression of positive regulators [63]. Given that these regulators did not exhibit significant alteration in *Gata6* cKO hindlimb buds, the preaxial polydactyly phenotype in *Gata6* cKO limbs is unlikely to be induced through these genes. A recent study suggested that *Gata6* represses *Shh* in the limb through binding to ZRS [38]. Our data is consistent with this report, and demonstrated that *Shh* and its targets are ectopically expressed in *Gata6* cKO hindlimb buds at E11.5. Restoration of normal expression pattern of *Gli1* and *Ptch1* in *Gata6* cKO; *Shh*^*+/-*^ hindlimbs also supports the idea that *Gata6* is upstream of *Shh*.

The second role is repressing ectopic Hedgehog signaling by enhancing repressor function of *Gli3*. Ectopic *Shh* expression in the *Gata6* cKO background affects data interpretations; however, compound heterozygous mutant analyses could enable separate analysis of the two mechanisms and support the second mechanism. Previous studies have shown *Gli3* to genetically interact with other genes during limb development. Studies on *Hox* genes suggested that the *Gli3*^*-/-*^ polydactyly phenotype is mediated by *Hoxd9* and *Hoxd10* [29, 64]. In addition, it has been shown that polydactyly of *Gli3*^*-/-*^ limbs becomes milder on the *Alx4*^*-/-*^ or *Zic3*^*-/-*^ background [30, 31], which suggested that the *Gli3*^*-/-*^ polydactyly phenotype requires *Alx4* or *Zic3*. In contrast to these reports, loss of one allele of *Gata6* enhanced the polydactyly phenotype of *Gli3*^*+/-*^ hindlimbs. Therefore, unlike previous genetic studies, our study identified *Gata6* as a negative factor for polydactyly development. Given that GLI3R prevents extra-digit formation in the anterior mesenchyme [55], our results suggest that *Gata6* cooperates with GLI3R activities.

It is believed that d1 develops in a *Shh*-independent manner, while development of d2-d5 requires *Shh* [5, 6, 10, 11]. Genetic manipulation of *Gli3* in mice provided evidence that high levels of GLI3R in the anterior of limb buds is necessary for proper d1 development and ensuring pentadactyly [24, 55, 65]. Expression pattern of *Pax9*, which requires high levels of GLI3R [56], indicates that *Gata6* contributes to GLI3R activities in the anterior of hindlimb buds. In particular, *Pax9* was undetectable in *Tcre; Gata6*^*+/fl*^; *Gli3*^*+/-*^ hindlimb buds, similar to *Gata6* cKO and *Gli3*^*-/-*^ hindlimb buds. These altered expression pattern of *Pax9* correlates with ectopic digit condensation and preaxial polydactyly, and further supports the idea that *Gata6* cooperate with *Gli3* for proper GLI3R activities in the anterior of hindlimb buds.

How does *Gata6* cooperate with *Gli3*? Our data support the idea that GATA6 physically interacts with GLI3R, facilitates the nuclear localization of GLI3R, and enhances the repressor activities of GLI3R. Reduced nuclear GLI3R localization in *Gata6* cKO hindlimb supports the idea that this interaction-mediated nuclear GLI3R localization would also occur in vivo. A recent study showed that *Gata4*, *5*, *and 6* can repress *Gli*-dependent reporter activation in vitro [66]. This study suggested that GATA inhibits SHH-dependent GLI activator function by protein interaction in the chick presomitic mesoderm. Based on this report and our study, GATA might modulate both GLI3R (this study) and SHH-dependent GLI activator [66] in a context-dependent manner. Since expression of *Gata* genes is reported in other *Gli3*-positive developing tissues, such as the branchial arch, somite and central nervous system [16, 67, 68], *Gata* regulation of GLI3R might be a shared mechanism during the development of other organs.

## Materials and Methods

### Ethics statement

Animal breeding was performed according to the approval by the Institutional Animal Care and Use Committee of the University of Minnesota. Compressed CO_2_ gas from a cylinder followed by cervical dislocation was the methods of euthanasia for mice. All efforts were made to minimize suffering.

### Mouse lines and embryo

The mouse lines for *Gata6*^*fl*^ [41], *Gli3*^*-*^ [69] and *Tcre* [42] were maintained on a mixed genetic background. Skeletal preparation was done as previously published [70]. Whole mount in situ hybridization was done as previously published [13].

### Expression constructs

The full-length human GATA6 construct and the human GLI3 construct were published [31, 71]. The GLI3R construct was generated by deleting the 3’ part of full-length cDNA, and cloned into 3xFlag CMV7. GATA6 deletion constructs were generated by PCR-based cloning and cloned in pcDNA3.1 or pCS2.

### Immunofluorescence and confocal imaging for GLI3R localization

For in vitro analysis, cells were fixed with 4% PFA for two hours at room temperature, washed with PBS and stained with anti-Flag (Sigma, M2, F3165, dilution 1:500) and anti-Myc tag (Abcam, ab9106, dilution 1:500) antibodies. For in vivo analysis, embryos were fixed for two hours in 4% PFA at 4C, washed with cold PBS, and cryosectioned with the OCT compound at 14 μm thickness. Sections were stained according to a standard procedure [13] without heat-induced epitope retrieval. Anti-GATA6 (R&D Systems, AF1700, dilution 1:400) and anti-GLI3R (Clone 6F5, dilution 1:200) [15, 72] were used. Alexa fluorophore-labelled secondary antibodies were obtained from Invitrogen (1:1000 dilution). Fluorescent confocal images were obtained by using Zeiss LSM 710 laser scanning microscope system (Carl Zeiss Microscopy), and analyzed using ZEN2009 software (Carl Zeiss Microscopy).

For subcellular localization analysis in vitro, images were acquired form six arbitrary areas from two plates. Nuclear/cytoplasmic localization of GLI3R and GATA6 was blindly evaluated in cells that were doubly transfected with GLI3R and GATA6 (or its mutants) except for samples that are transfected with GLI3R alone. For in vivo samples, nuclear/cytoplasmic localization of GLI3R was evaluated similarly in the anterior-proximal domain where GATA6 signals in wild-type hindlimb buds were detected. In *Gata6* cKO embryos, the anterior-proximal domain, similar to wild-type embryos, was selected for GLI3R subcellular localization. The quantification was performed similar to in vitro samples.

### GATA6 localization in hindlimb buds

In order to clarify GATA6 localization in hindlimb bud mesenchyme, GATA6 was simultaneously detected with limb bud mesenchyme markers, such as FGF10 or DUSP6. Wild-type embryos were fixed, washed and cryosectioned as described above. Sections were simultaneously stained by anti-GATA6 (R&D AF1700 or Cell Signaling #5851, dilution 1:1,600) and anti-FGF10 (Santa Cruz, sc-7917, dilution 1:100) or anti-DUSP6 (Sigma, Clone 3G2, dilution 1:200). Sections were reacted with Alexa fluorophore-labelled secondary antibodies, and fluorescent signals were detected by Zeiss LSM 710 according to a standard procedure [13].

### Luciferase reporter assay

NIH3T3 cells in 48-well plates were transfected with the 12xGLI-binding site-TK minimum promoter-luciferase [31] with pRL-TK, *GATA6* and/or GLI3R expression constructs by using Fugene6 (Promega). Forty hours after transfection, cells were subjected to analysis using the Dual-Luciferase Reporter Assay System (Promega). Experiments were performed in triplicate, and statistical significance was analyzed by One-way ANOVA followed by the Tukey’s comparison.

### Co-immunoprecipitation assay and nuclear GLI3R detection

HEK293T cells were transfected with expression constructs by using the standard calcium phosphate method. Cell lysates, prepared after two days, were passed through 25 gauge syringes to ensure protein extraction from the nucleus, and co-immunoprecipitation assays were performed by using Dynabeads protein G (Invitrogen) and anti-Flag (Sigma, M2, F3165, 2μg) or anti-Myc tag (Abcam, ab9106, 1 μg) antibodies. Proteins were resolved by SDS-PAGE, transferred to PVDF membranes (Millipore, MA, USA), reacted with anti-Myc tag or anti-Flag antibodies, followed by HRP goat anti-mouse or rabbit IgG, and a chemiluminescence detection.

For co-immunoprecipitation assays with in vivo samples, hindlimb buds were collected from wild-type embryos at E10.25–10.5. After pooling, the samples were lysed and subjected to co-immunoprecipitation procedures [73] using anti-GATA6 (Cell Signaling, #5851) and Dynabeads protein G. The protein complex was eluted, and detected by Western using anti-GLI3 (R&D Systems, AF3690, dilution 1:100) and the PicoLUCENT PLUS HRP detection kit (G-Bioscience) according to the manufacturer’s instructions.

For nuclear GLI3R detection by Western, anterior one third of hindlimb buds at E10.25–10.5 were collected, and the nuclear fraction was prepared after dissociating cells by using the NE-PER kit (Thermo Fischer) according to the manufacturer’s instructions. The nuclear extracts were analyzed by Western using anti-GLI3 (R&D Systems, AF3690) and anti-Histone H3 (Abcam, ab-1791).

## Supporting Information

S1 Fig Expression pattern of *Shh* and its target genes at E11.5. In situ hybridization of indicated genes in hindlimb buds of wild type (**A-E**) and *Gata6* cKO (**F-J**) at E11.5.
(TIFF)

S2 Fig Expression pattern of negative regulators of *Shh* expression at E10.5. In situ hybridization of indicated genes in hindlimb buds of wild type (**A-D**) and *Gata6* cKO (**E-H**) at E10.5.
(TIFF)

S3 Fig Images of subcellular localization of GLI3R, GATA6 and GATA6 mutants. HEK293 cells were transfected with GLI3R and indicated forms of GATA6 (wild type or deletion mutants). Panels show staining by anti-Myc antibodies (GATA6), anti-Flag antibodies (GLI3) or merged images.
(TIF)

S4 Fig GATA6 localization in hindlimb buds. (**A**) *Gata6* mRNA expression. *Gata6* is expressed in the anterior proximal region of hindlimb buds (arrowhead). (**B, C**) Co-immunofluorescence of GATA6 with DUSP6 (B) or FGF10 (C). Transverse sections were stained with antibodies for indicated proteins. Dotted areas indicate hindlimb buds. Shown are sections corresponding to the anterior region. GATA6 is expressed in the ventral side of anterior mesenchyme (white arrows). d: dorsal side, v: ventral side.
(TIF)

S1 Table Number of *Gata6* mutants using the *Tcre* deleter. Embryos at E13.5–15.5 were collected. The breeding pairs are *Gata6*^*fl/fl*^ and *Tcre*^*Tg/Tg*^; *Gata6*^*+/fl*^.
(DOCX)

S2 Table Number of forelimbs with indicated phenotypes at E14.5–16.5. Embryos at E14.5–16.5 were collected and scored.
(DOCX)
