# Supplementary figures and images for: Gata6-Dependent GLI3 Repressor Function is Essential in Anterior Limb Progenitor Cells for Proper Limb Development
Source: PLoS Genet. 2016 Jun 28;12(6):e1006138. doi: 10.1371/journal.pgen.1006138 (PMC4924869; doi:10.1371/journal.pgen.1006138)

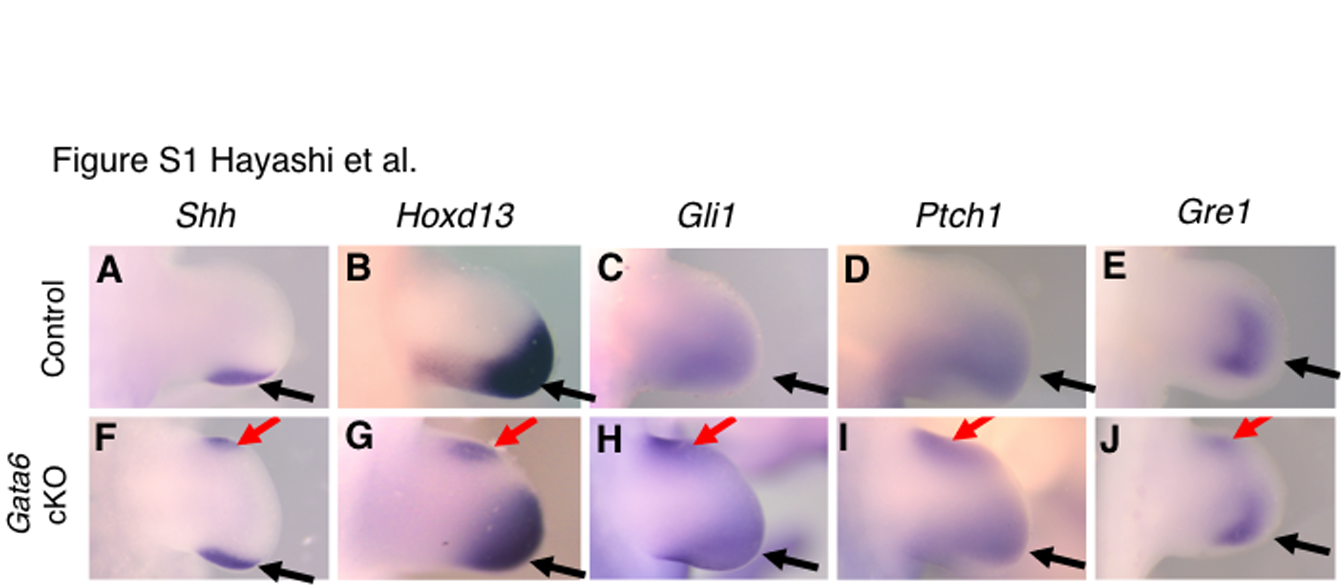

Supplement: S1 Fig — In situ hybridization of indicated genes in hindlimb buds of wild type (A-E) and Gata6 cKO (F-J) at E11.5. (TIFF) [file pgen.1006138.s001.tiff]

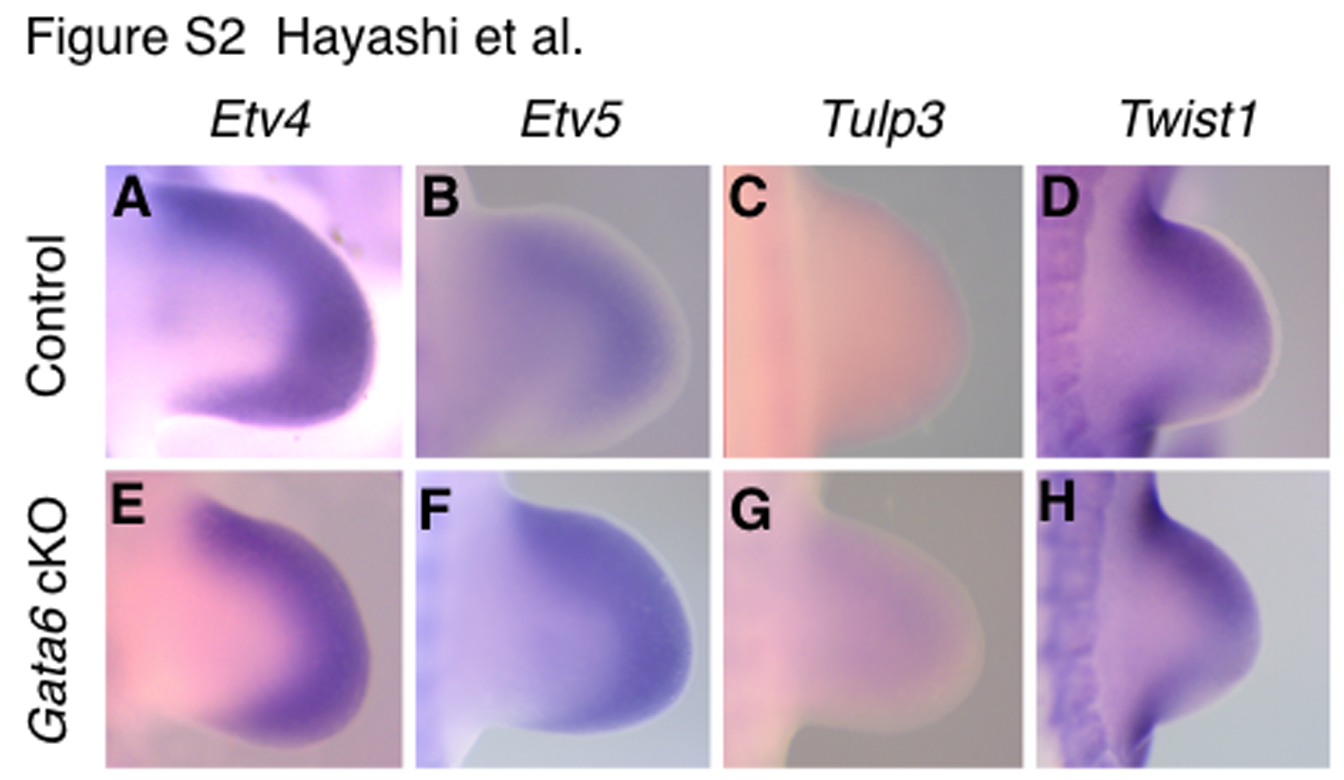

Supplement: S2 Fig — In situ hybridization of indicated genes in hindlimb buds of wild type (A-D) and Gata6 cKO (E-H) at E10.5. (TIFF) [file pgen.1006138.s002.tiff]

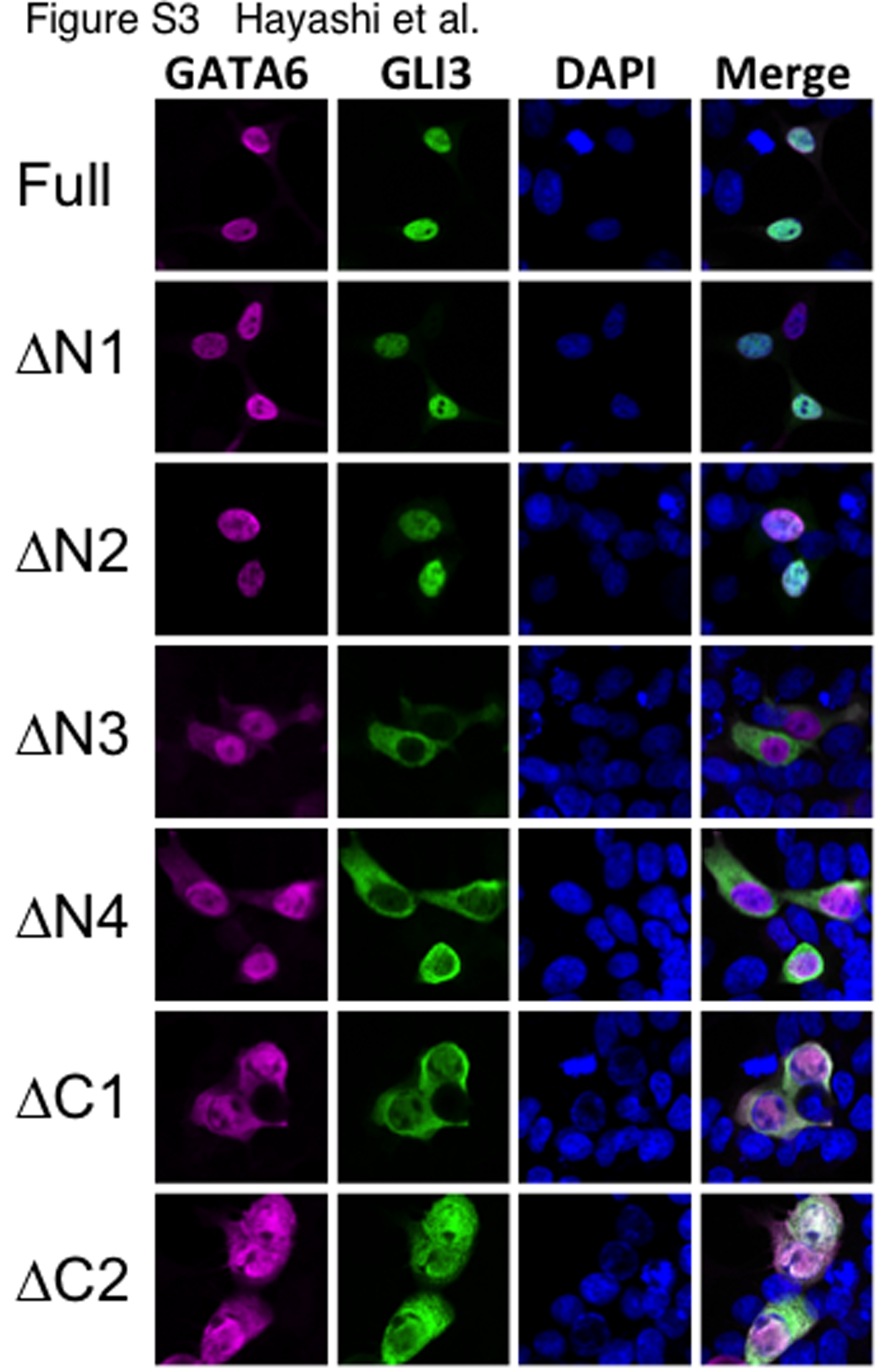

Supplement: S3 Fig — HEK293 cells were transfected with GLI3R and indicated forms of GATA6 (wild type or deletion mutants). Panels show staining by anti-Myc antibodies (GATA6), anti-Flag antibodies (GLI3) or merged images. (TIF) [file pgen.1006138.s003.tif]

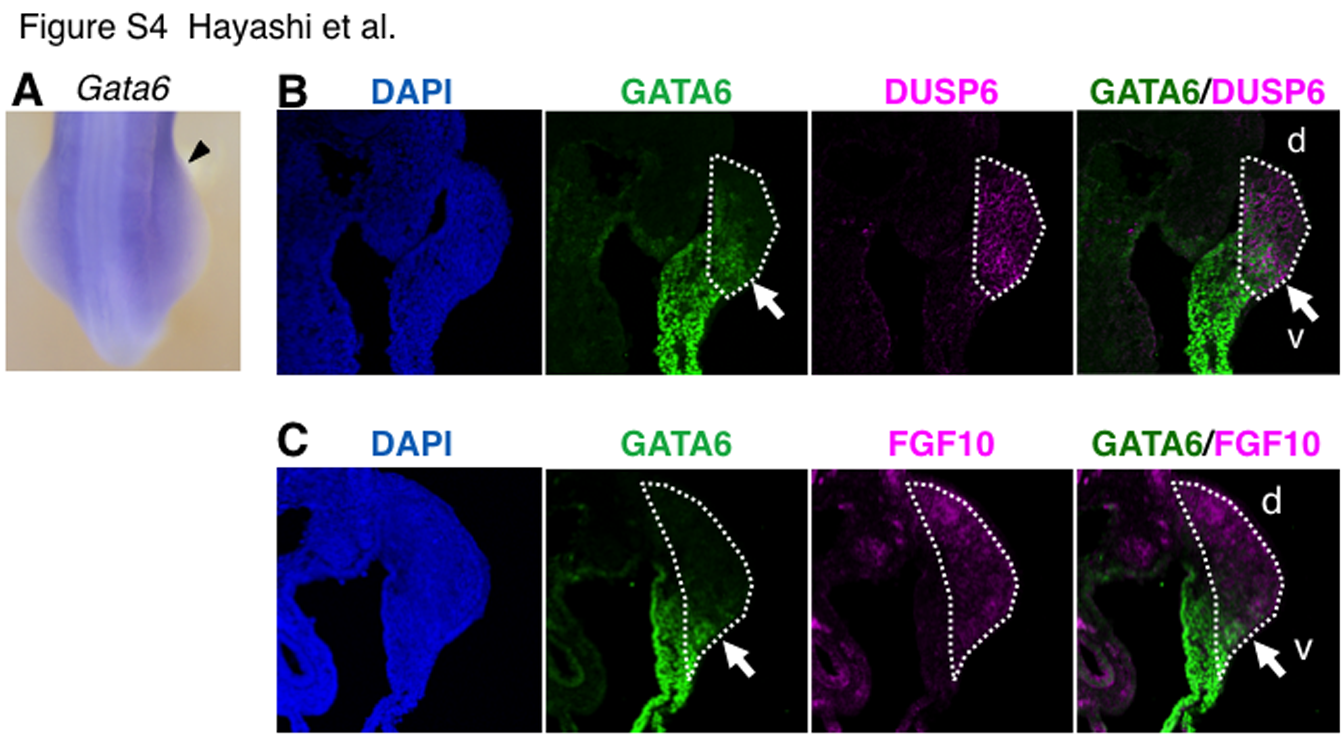

Supplement: S4 Fig — (A) Gata6 mRNA expression. Gata6 is expressed in the anterior proximal region of hindlimb buds (arrowhead). (B, C) Co-immunofluorescence of GATA6 with DUSP6 (B) or FGF10 (C). Transverse sections were stained with antibodies for indicated proteins. Dotted areas indicate hindlimb buds. Shown are sections corresponding to the anterior region. GATA6 is expressed in the ventral side of anterior mesenchyme (white arrows). d: dorsal side, v: ventral side. (TIF) [file pgen.1006138.s004.tif]
