# Supplementary material for: Gata6-Dependent GLI3 Repressor Function is Essential in Anterior Limb Progenitor Cells for Proper Limb Development
Source: PLoS Genet. 2016 Jun 28;12(6):e1006138. doi: 10.1371/journal.pgen.1006138 (PMC4924869; doi:10.1371/journal.pgen.1006138)
Supplement: S1 Table — Embryos at E13.5–15.5 were collected. The breeding pairs are Gata6fl/fl and TcreTg/Tg; Gata6+/fl. (DOCX) [file pgen.1006138.s005.docx]

S1 Table

Number of *Gata6* mutants using the *Tcre* deleter

| Breeding pair | Total number  of embryos | Number of cKO | cKO with  polydactyly in hindlimbs | Absorbed or dead before E12.5 |
| --- | --- | --- | --- | --- |
| *Tcre; Gata6^+/fl^*  *and Gata6^fl/fl^* | 86 | 12 | 12 | 27 |
