# Supplementary material for: Gata6-Dependent GLI3 Repressor Function is Essential in Anterior Limb Progenitor Cells for Proper Limb Development
Source: PLoS Genet. 2016 Jun 28;12(6):e1006138. doi: 10.1371/journal.pgen.1006138 (PMC4924869; doi:10.1371/journal.pgen.1006138)
Supplement: S2 Table — Embryos at E14.5–16.5 were collected and scored. (DOCX) [file pgen.1006138.s006.docx]

S2 Table

Number of forelimbs with indicated phenotypes at E14.5-16.5

| Genotype | Normal digits | Small cartilage condensation at the tip of d1 | Incomplete extra d1 | Extra d1 |
| --- | --- | --- | --- | --- |
| Wild type | 140/140 (100%) | 0/140 | 0/140 | 0/140 |
| *Gli3^+/-^* | 0/18 (0%) | 18/18 (100%) | 0/18 (0% | 0/18 (0%) |
| *Tcre; Gata6^+/fl^* | 66/66 (100%) | 0/66 (0%) | 0/66 (0%) | 0/66 (0%) |
| *Tcre; Gata6^+/fl^; Gli3^+/-^* | 4/54 (7.4%) | 14/54 (25.9%) | 23/54 (42.6%) | 13/54 (24.0%) |

Embryos at E14.5-16.5 were collected and scored.
